# Diagnostic Salivary Gland Biopsy in Pediatric Eosinophilic Granuolmatosis with Polyangiitis

**DOI:** 10.1002/oto2.70149

**Published:** 2025-08-14

**Authors:** Keiko Fox, Nicole Wershoven, Soham Roy, Jeremy D. Prager

**Affiliations:** ^1^ School of Medicine University of Colorado Aurora Colorado USA; ^2^ Department of Otolaryngology–Head and Neck Surgery University of Colorado Aurora Colorado USA; ^3^ Department of Otolaryngology Children's Hospital Colorado Aurora Colorado USA

**Keywords:** ANCA‐associated vasculitis, eosinophilia, eosinophilic granulomatosis with polyangiitis (EGPA), pediatric vasculitis, salivary gland biopsy

Eosinophilic granulomatosis with polyangiitis (EGPA) is a rare anti‐neutrophil cytoplasmic antibody (ANCA) associated vasculitis which progresses from allergic rhinosinusitis and asthma to eosinophilia and eventual systemic vasculitis.^1^, ^2^, ^3^ Hypereosinophilia, vascular inflammation, and granulomatosis are hallmarks,^2^ impacting multiple systems, including renal, pulmonary, and otolaryngologic.^1^, ^2^

Mean age at diagnosis is 49 years.^3^ Pediatric cases are rare;^2^ incidence of any ANCA vasculitis is approximately 0.24 per 100,000 children annually.^4^ Literature on pediatric EGPA remains limited.^1^, ^2^ Pediatric presentations are frequently atypical, often lacking childhood vasculitis, complicating diagnosis using adult‐based criteria.^1^ Worse longterm outcomes in pediatric EGPA underscore need for improved diagnostic strategies.

Ear, nose, and throat (ENT) symptoms occur in 60% to 80% of patients,^2^ including asthma, allergic rhinitis, and chronic rhinosinusitis.^3^ In children, nasal polyps, chronic otitis, and salivary gland swelling are particularly common.^1^ Early sinonasal manifestations mean otolaryngologists may encounter patients early in the disease course.

Biopsy is recommended in suspected EGPA.^2^, ^5^ However, typical ENT sites like sinonasal mucosa and polyps yield diagnostic samples less reliably than other organs.^2^ Diagnosis may thus be limited by invasiveness of confirmatory sampling. Salivary gland biopsy, common in IgG4‐related disease, appears rare in EGPA. We report a pediatric case wherein submandibular gland biopsy demonstrated diagnostic EGPA histopathology.

## Case Description

A 14‐year‐old male with history of allergic rhinosinusitis presented to the Children's Hospital Colorado (CHCO) ED for 2 months of sinusitis, cough, fatigue, weight loss, nasal congestion, new‐onset fever, periorbital edema, and tender cervical lymphadenopathy. Initial steroid and antibiotic management provided minimal relief; diagnosis was viral URI.

Two months later, symptoms persisted, and weight loss totaled 16 pounds, attributed to jaw pain from tender submandibular masses. At CHCO's otolaryngology clinic, labs revealed leukocytosis, eosinophilia, elevated inflammatory markers, positive c‐ANCA and Anti‐MPO IgG, elevated IgE and C3, and broad aeroallergen positivity. p‐ANCA, RF, ANA, and infectious studies were negative.

**Figure 1:**
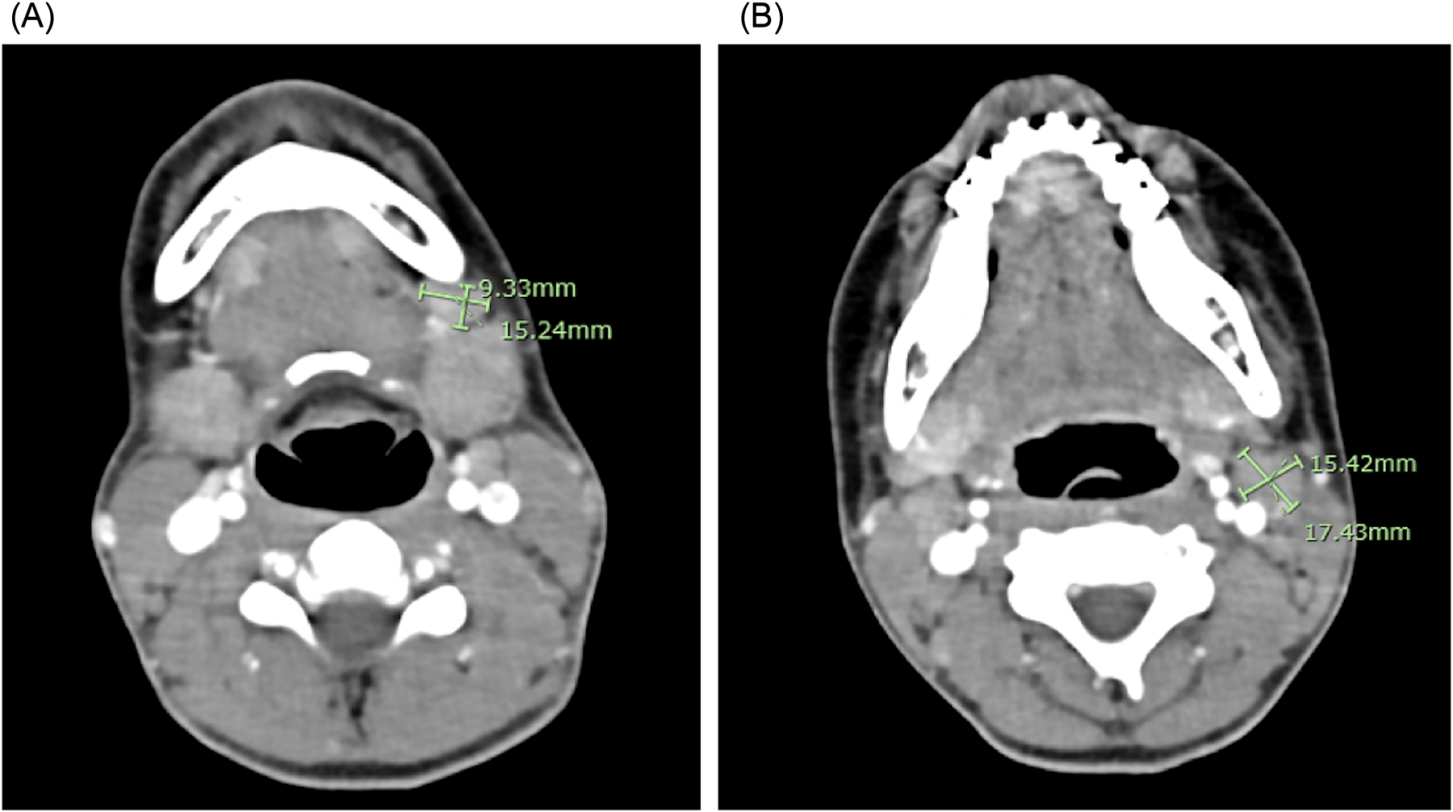
Contrast CT neck/chest. (A) Bilateral submandibular masses with largest left‐sided mass measuring 15 × 9 mm. (B) Mildly enlarged bilateral jugulodigastric lymph nodes with left node measuring 17 × 15 mm.

The Neck/Chest CT radiology report described what were believed to be normal‐appearing parotid and submandibular glands with enlarged submandibular and jugulodigastric “nodes” (Figure 1) and noncalcified, noncavitating bibasilar lung nodules.

Following further examination, otolaryngology providers’ suspicion that the “nodes” were enlarged submandibular glands rather than lymph nodes prompted excisional and incisional biopsy of the especially enlarged left level 2A lymph node and submandibular gland, respectively, plus bronchoscopy and bronchoalveolar lavage.

Lymph node biopsy revealed acute lymphadenitis and sinus histiocytosis. Bronchoscopy found thick, white secretions in the trachea, carina, and lungs. Friable, nodular mucosa was identified throughout the lungs and middle and lower trachea. Cytology demonstrated 4% eosinophils. Culture and gram stain found no organisms.

**Figure 2:**
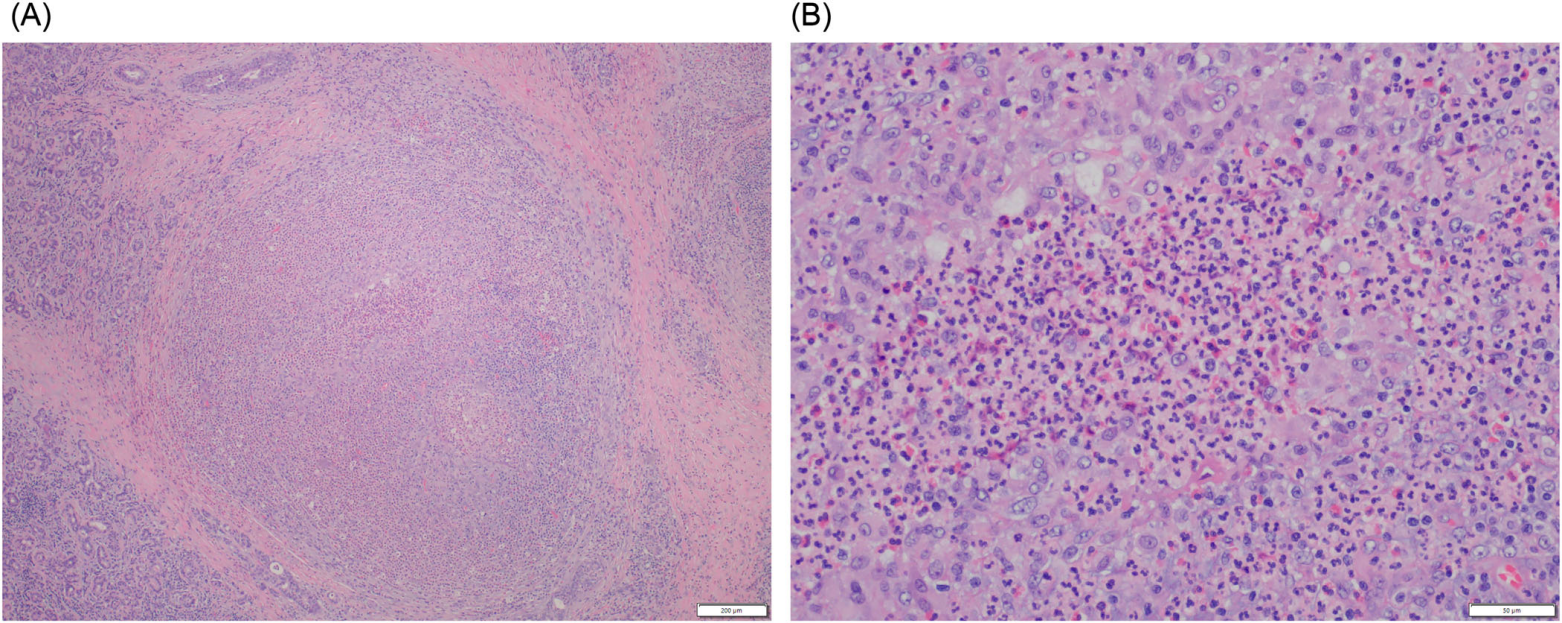
Left submandibular salivary gland specimen histology. Hematoxylin and eosin stain demonstrating eosinophil‐rich sialadenitis/sialodochitis with periductal granuloma formation and fibrosis. Original magnification ×4 (A) and ×20 (B).

Submandibular gland biopsy identified eosinophil‐rich parenchymal and ductal inflammation, periductal granuloma, and interlobular fibrosis (Figure 2). IgG4:IgG ratio was ~20%. Though vasculitis was absent, EGPA diagnosis was reached.

The patient received prednisone and inhaled/intranasal steroids. Two months later, CHCO's allergy clinic introduced mepolizumab for steroid weaning. The regimen was well tolerated; symptoms resolved and inflammatory markers normalized. Now 5 months post‐diagnosis, he continues utilizing inhaled steroids and tapering prednisone.

## Discussion

Existing EGPA diagnostic criteria have been criticized for excluding pediatric presentations; here, absent vasculitis made diagnosis challenging. However, following otolaryngologic evaluation, salivary gland biopsy findings yielded evidence needed to fulfill American College of Rheumatology diagnostic criteria, aligning with reports that pediatric EGPA is often diagnosed in the eosinophilic phase.^1^

Timely diagnosis remains challenging, particularly when differentiating from other eosinophilic conditions.^5^ Regardless, as pediatric EGPA demonstrates higher relapse rates and worse outcomes relative to adult disease, early diagnosis remains essential.^1^

Salivary gland involvement is not classic of EGPA; however, eosinophilia can affect nearly any organ, even in early stages.^3^, ^5^ Literature supports diagnostic value of biopsy in EGPA, with 1 study reporting 91.6% confirmation through histopathology.^5^ An accessible biopsy site, salivary glands provide useful samples for distinguishing EGPA from other eosinophilic disorders.^5^ In select cases when EGPA is being considered and salivary gland inflammation is present, salivary gland biopsy may offer a less invasive, valuable diagnostic tool.

## Conclusion

This case highlights a unique approach in pediatric EGPA. Submandibular gland biopsy—prompted by otolaryngologic evaluation of large, tender, extremely firm submandibular glands—yielded diagnostic histopathology despite an atypical presentation lacking vasculitis. This case underscores the potential of salivary gland biopsy in facilitating early detection in a population facing significant morbidity in the context of lesser‐understood disease.

## Author Contributions

**Keiko Fox**, writing—original draft, writing—review and editing; **Nicole Wershoven**, writing—original draft, writing—review and editing; **Soham Roy**, writing—review and editing, supervision; **Jeremy D. Prager**, writing—review and editing, supervision.

## Disclosure

### Competing interests

None.

### Funding source

None.

## Ethics Approval

This submission was determined to be exempt by the Colorado Multiple Insititutional Review Board (COMIRB# 24‐2394).
